# Stem Cell Antigen-1 in Skeletal Muscle Function

**DOI:** 10.1371/currents.md.411a8332d61e22725e6937b97e6d0ef8

**Published:** 2013-08-15

**Authors:** Harold S. Bernstein, Tahmina Samad, Sompob Cholsiripunlert, Saami Khalifian, Wenhui Gong, Carissa Ritner, Julian Aurigui, Vivian Ling, Karlijn J. Wilschut, Stephen Bennett, Julien Hoffman, Peter Oishi

**Affiliations:** University of California San Francisco, San Francisco, California, USA; University of California San Francisco, San Francisco, California, USA; University of California San Francisco, San Francisco, California, USA; University of California San Francisco, San Francisco, California, USA; University of California San Francisco, San Francisco, California, USA; University of California San Francisco, San Francisco, California, USA; University of California San Francisco, San Francisco, California, USA; University of California San Francisco, San Francisco, California, USA; University of California San Francisco, San Francisco, California, USA; University of California Davis, Davis, California, USA; University of California San Francisco, San Francisco, California, USA; University of California San Francisco, San Francisco, California, USA

## Abstract

Stem cell antigen-1 (Sca-1) is a member of the Ly-6 multigene family encoding highly homologous, glycosyl-phosphatidylinositol-anchored membrane proteins. Sca-1 is expressed on muscle-derived stem cells and myogenic precursors recruited to sites of muscle injury. We previously reported that inhibition of Sca-1 expression stimulated myoblast proliferation in vitro and regulated the tempo of muscle repair in vivo. Despite its function in myoblast expansion during muscle repair, a role for Sca-1 in normal, post-natal muscle has not been thoroughly investigated. We systematically compared Sca-1-/- (KO) and Sca-1+/+ (WT) mice and hindlimb muscles to elucidate the tissue, contractile, and functional effects of Sca-1 in young and aging animals. Comparison of muscle volume, fibrosis, myofiber cross-sectional area, and Pax7+ myoblast number showed little differences between ages or genotypes. Exercise protocols, however, demonstrated decreased stamina in KO versus WT mice, with young KO mice achieving results similar to aging WT animals. In addition, KO mice did not improve with practice, while WT animals demonstrated conditioning over time. Surprisingly, myomechanical analysis of isolated muscles showed that KO young muscle generated more force and experienced less fatigue. However, KO muscle also demonstrated incomplete relaxation with fatigue. These findings suggest that Sca-1 is necessary for muscle conditioning with exercise, and that deficient conditioning in Sca-1 KO animals becomes more pronounced with age.

## INTRODUCTION

In response to skeletal muscle damage, resident myogenic progenitors undergo activation to form a pool of proliferating myoblasts. These mononuclear myoblasts differentiate and fuse, forming multinucleated myocytes, which repair or replace the damaged tissue 1 ^,^ 2 ^,^ 3 . This programmed series of events is essential to maintaining tissue homeostasis during exercise and aging, and to ensuring recovery from muscle trauma 4 . While the balance between myoblast proliferation and differentiation is critical to muscle repair, its regulation is incompletely understood.

We previously identified Stem cell antigen-1 (Sca-1; also known as Ly-6A/E) during an expression screen to identify genes regulating myoblast cell cycle withdrawal during differentiation 5 . Sca-1 is a member of the Ly-6 multigene family encoding a number of highly homologous, glycosyl-phosphatidylinositol (GPI)-anchored surface membrane proteins, and is widely used as a marker of murine hematopoietic stem cells 6 ^,^ 7 ^,^ 8. Beyond its role as a stem cell marker, it has been shown that overexpression of Sca-1 inhibits proliferation of CD4^+^ T-cells (9), as well as differentiation of hematopoietic stem cells 6 ^,^ 10 ^,^ 11 ^,^ 12 . Sca-1^-/-^ mice are viable, however, they exhibit immune and hematopoietic defects 6 ^,^ 10 ^,^ 11 ^,^ 12 . Specifically, these mice demonstrate a lymphocytosis and thrombocytopenia, and isolated Sca-1^-/-^ T-cells undergo prolonged hyperproliferation with stimulation *in vitro* 11 . Consistent with a role in progenitor cell maintenance, Sca-1-null animals have a reduced ability to re-populate bone marrow after serial transplantation 6 ^,^ 12 and develop age-related failure of osteogenesis 10 .

Sca-1 also is expressed on the surface of muscle-derived stem cells 13 ^,^ 14 and myogenic precursors recruited to sites of skeletal or cardiac muscle injury 13 ^,^ 15 ^,^ 16 ^,^ 17 ^,^ 18 . We previously reported that inhibition of Sca-1 expression by antisense or Sca-1 interference with blocking antibodies stimulated myoblast proliferation and delayed myoblast fusion *in vitro* 19. Subsequently, others observed sustained proliferation in Sca-1^-/-^ myoblasts cultured *ex vivo* 20 . Using a myonecrotic injury model in Sca-1^-/-^ and Sca-1^+/+^ mice, we then showed that Sca-1 regulates the tempo of muscle repair by controlling the balance between proliferation and differentiation of activated myoblasts 21 .

Despite its function in stem cell proliferation in general, and more specifically in myoblast expansion during secondary myogenesis, a role for Sca-1 in normal, post-natal muscle function has not been apparent. To explore this, we undertook the systematic comparison of Sca-1^-/-^and Sca-1^+/+^mice and hindlimb muscles to elucidate the tissue, mechanical, and functional effects of Sca-1 in young adult and aging animals.

## MATERIALS AND METHODS

***Animals.*** All animal procedures were approved by the Institutional Animal Care and Use Committee at the University of California, San Francisco, in accordance with the Association for Assessment and Accreditation of Laboratory Animal Care International guidelines. Mice heterozygous at the Sca-1 locus were graciously provided by Patrick Flood (University of North Carolina) 11 , and backcrossed to BALB/c strain for ten generations. Sca-1^+/-^ littermates were bred to homozygosity. Genotypes were confirmed for all experimental animals by Southern blot analysis, as previously described 21 . All experiments were performed on 12-16 week-old or 47-50 week-old female mice. Wild type (Sca-1^+/+^) female BALB/c littermates from heterozygous matings were used as controls in all experiments.

***Muscle volume analysis.*** Animals were euthanized with intraperitoneal injection of pentobarbital followed by cervical dislocation. Following euthanization, skinned hindlimbs were fixed in 10% paraformaldehyde, then dehydrated in ethanol and embedded in paraffin. Hindlimbs were sectioned at 5 μm perpendicular to the myofiber through the length of the limb. A series of every tenth section was selected and processed for staining. Sections were deparaffinized and stained with hematoxylin and eosin to identify tissue architecture. Using accepted anatomic boundaries, the relevant muscle was traced at low power (4X) using the live image generated with a Zeiss Axiovision fluorescent microscope (Carl Zeiss; Thornwood, NY) equipped with a Hamamatsu Orca2 digital camera (Hamamatsu; Bridgewater, NJ). Volume estimates were calculated using the Cavalieri principle 22 and contours traced at low power. Both hindlimbs from at least 3 animals were analyzed for each genotype/age.

***Myofiber CSA and perimeter analysis.*** Tissue sections were prepared as described above. A series of every tenth section was selected and processed for staining. Sections were deparaffinized in xylene and dehydrated with ethanol before antigen retrieval with proteinase K for 10 minutes at room temperature (RT). After washing out proteinase K, sections were incubated with 3% H_2_O_2_ in methanol for 10 min at RT, Rodent Block M (BioCare; Concord, CA) for 30 min at RT, DAKO antibody diluent (DAKO; Carpinteria, CA) for 30 min at RT, rabbit anti-laminin (Sigma-Aldrich; St. Louis, MO) at 1:100 dilution in DAKO antibody diluent (DAKO; Carpinteria, CA) overnight at 4°C, then washed with phosphate-buffered saline. To develop peroxidase, sections were incubated with Rabbit HRP Polymer (BioCare; Concord, CA), then DAB substrate (BioCare; Concord, CA) according to the manufacturer’s instructions. Sections were counterstained with hematoxylin before dehydration with ethanol (80, 90, 100%). To quantitate myofiber cross-sectional area (CSA) and perimeter within a section, ≥300 myofibers within a centrally located field (20X; ~300,000 µm^2^) within the tibialis anterior, extensor digitorum longus, or soleus muscle were analyzed using Stereo Investigator software (Microbrightfield; Williston, VT). Both hindlimbs from at least 3 animals were analyzed for each genotype/age.

***Pax7 analysis. ***Paraffin-embedded tissue sections were prepared as described above. Sections were incubated with Rodent Block M (BioCare; Concord, CA) for 30 min at RT, blocking buffer (2% goat serum, 1% bovine serum albumin, 0.1% fish gelatin, 0.1% Triton X-100, 0.1% glycine) for 30 min at RT, 10 µg/ml Alex488-conjugated anti-Pax7 (R&D Systems; Minneapolis, MN) in blocking buffer without glycine for 60 min at RT, then washed with phosphate-buffered saline, counterstained with DAPI, and dehydrated with ethanol (70, 80, 90, 100%). Immunostained sections were imaged by randomly placing the 20X objective within the tibialis anterior muscle within the section. Following imaging, the field was manually moved a fixed distance of approximately 600 µm in the horizontal and then vertical axis, resulting in 4–6 counting images per section. DAPI^+^Pax7^+^ nuclei localized beneath the basal lamina of myofibers were confirmed at 60X magnification, then counted and expressed as a percentage of total DAPI^+^ nuclei. Both hindlimbs from at least 3 animals were analyzed for each genotype/age. ******

***Arteriole number and CSA analysis. ***Tissue sections were prepared as described above. Sections were incubated with Rodent Block M (BioCare; Concord, CA) for 30 min at RT, DAKO antibody diluent (DAKO; Carpinteria, CA) for 30 min at RT, rat anti-CD31 (BioCare; Concord, CA) at 1:25 dilution in DAKO antibody diluent (DAKO) overnight at 4°C, then washed with phosphate-buffered saline. To develop peroxidase, sections were incubated with Rat HRP Polymer (BioCare; Concord, CA) followed by DAB substrate (BioCare; Concord, CA) according to the manufacturer’s instructions. After rinsing with phosphate-buffered saline, sections were then re-blocked with Rodent Block M (BioCare; Concord, CA) for 30 min at RT and DAKO antibody diluent (DAKO; Carpinteria, CA) for 30 min at RT, and incubated with mouse anti-SMA (BioCare; Concord, CA) at 1:50 dilution for 60 min at RT. Sections were then incubated with Mouse AP Polymer (BioCare; Concord, CA) and developed with Vulcan Red (BioCare; Concord, CA) according to the manufactorer’s instructions. Sections were counterstained with hematoxylin before dehydration with ethanol (70, 80, 90, 100%). Immunostained sections were imaged using the 4X objective. Arteriole number was calculated as the total number of SMA^+^CD31^+^ vessels per section. Each vessel was confirmed at 40X. To quantitate arteriolar CSA, all identified arterioles were analyzed using Stereo Investigator software (Microbrightfield; Williston, VT). Both hindlimbs from at least 3 animals were analyzed for each genotype/age.

***Fibrosis. ***Tissue sections were prepared as described above. A series of every tenth section was selected and processed for staining. Sections were deparaffinized in xylene and dehydrated with ethanol before staining with aniline blue, hematoxylin, and scarlet acid fuchsin with Masson’s Trichrome 2000 stain kit (American MasterTech, Lodi, CA) according to the manufacturer’s instructions. Stained sections dehydrated with alcohol, cleared with xylene, and imaged using the 4X objective. To quantitate CSA of fibrosis, all stained areas were analyzed using Stereo Investigator software (Microbrightfield; Williston, VT). Both hindlimbs from at least 3 animals were analyzed for each genotype/age. Differences between genotype/age groups were tested for significance by one-way analysis of variance.

***Ki67 immunostaining. ***Tissue sections were prepared as described above. A series of every tenth section was selected and processed for staining. Sections were deparaffinized in xylene and dehydrated with ethanol before antigen retrieval with citrate buffer for 20 minutes at 37°C. After washing with phosphate-buffered saline, sections were incubated with 3% H_2_O_2_in methanol for 20 min at RT, Rodent Block M (BioCare; Concord, CA) for 30 min at RT, DAKO antibody diluent (DAKO; Carpinteria, CA) for 30 min at RT, rat anti-Ki67 (DAKO; Carpinteria, CA) at 1:10 dilution in DAKO antibody diluent (DAKO; Carpinteria, CA) overnight at 4°C, then washed with phosphate-buffered saline. To develop peroxidase, sections were incubated with Rat HRP Polymer (BioCare; Concord, CA), then DAB substrate (BioCare; Concord, CA) according to the manufacturer’s instructions. Sections were counterstained with hematoxylin then washed with phosphate-buffered saline, counterstained with DAPI, and dehydrated with ethanol. Immunostained sections were imaged by randomly placing the 20X objective within the section. Following imaging, the field was manually moved a fixed distance of approximately 600 µm in the horizontal and then vertical axis, resulting in 4–6 counting images per section. DAPI^+^Ki67^+^nuclei localized beneath the basal lamina of myofibers were confirmed at 60X magnification, then counted and expressed as a percentage of total DAPI^+^nuclei. Both hindlimbs from at least 3 animals were analyzed for each genotype/age. Differences between genotype/age groups were tested for significance by one-way analysis of variance.

***Exercise.*** To evaluate voluntary exercise, mice were allowed to run at will during normal light-dark cycles as established at the UCSF Laboratory Animal Resource Center. Distance and duration over consecutive 24h periods were recorded with individual FX10 cycle computers (E3 Cycling; Chapel Hill, NC). To evaluate forced exercise, mice were forced to run on each day over a 37d period on a Lafayette Mouse Forced Exercise Run/Walk Wheel System (Lafayette Instrument; Lafayette, IN) at 8 meters/min for five 10 min intervals with 30s rests between intervals. Over the next five 10 min intervals immediately following during that day’s exercise session, speeds were progressively increased until mice reached their maximum rate, as determined by the maximum speed (meters/min) at which mice continued to run on the wheel without falling off or hanging onto the wheel.

***Myomechanical analysis.*** Ex vivo muscle analysis was performed as previously described 23 . Following euthanization, the EDL muscle was extracted and placed in a bath containing Krebs Hensleit buffer within 15 min. The muscle was attached by opposing tendons to a DMT Model 820MS force transducer (Danish Myo Technology; Ann Arbor, MI) filled with Krebs Hensleit buffer prewarmed to 25^o^C and bubbled with O_2_/CO_2_ (95%/5%) for ≤15 min prior to use. During the mounting process, the muscle was only handled through the suture, without direct contact to the muscle to prevent damage to the muscle fiber. The muscle was stimulated by a Grass Model S48 square pulse electrical stimulator (Grass Technologies; West Warwick, RI) and the data analyzed and projected using a custom acquisition platform (ADInstruments PowerLab Data Acquisition System and LabChart software; ADInstruments; Colorado Springs, CO).

Twitch tension (P_t_) was recorded by first stretching the EDL muscle until there was no laxity in the muscle fiber. A square stimulation of 0.5 ms duration was used to induce twitch. The voltage was increase incrementally until maximal twitch tension was achieve and then the voltage was set at 20% above the maximum to induce a supramaximal stimulus (mean supramaximal stimulus was 40 volts). Optimum length of the muscle was determined by carefully stretching the muscle and recording the twitch response after square stimulation until maximal twitch was recorded. The muscle was left to equilibrate at the optimal length for 3 min before another supramaximal stimulus was applied and the output recorded as the twitch force. Tetanic tension (P_o_) was recorded by applying a train of supramaximal stimuli for 300 ms at 150 Hz.

Force-frequency fatigue was measured by exposing muscle to a supramaximal stimulus train of 3-5 pulses (300 msec duration separated by 3 sec) at successive frequencies (30, 60, 100 and 140 Hz), with 5 min intervals between stimulations. For a given frequency stimulus and muscle, the maximum pulse was chosen and then normalized relative to the peak response over all pulse frequencies. An average force-frequency diagram was then constructed from all normalized muscle responses. Force-frequency stimulation produces a maximum response at a given frequency that may fall off precipitously at other frequencies. The fatigue characteristic of a given group would be interpreted as a significant reduction in the peak relative to the other groups at the various frequencies.

Low-frequency time-fatigue was measured by supramaximal tetanic muscle stimulation at low frequency (60 Hz) for a duration of 300 msec, repeated every 3 seconds for a period of 10 min. The low-frequency time-fatigue curve produces a peak response at a given time that is followed by a multi-exponential decay in contraction force. Fatigue in a given group would be interpreted as a significant reduction in percent maximum contraction force over time, assuming that the different groups experienced the same average peak force at the onset. We sampled the percent of maximal force of contraction at given periods (0.5 , 1, 1.5, 2-10 min in 1 min increments).

Muscle mass, cross-sectional area, and length were measured after the fatigue analysis to avoid handling prior to analysis.

***Statistical analysis.***For volume, CSA, perimeter, and cell number analyses, differences between genotype/age groups were tested for significance by one-way analysis of variance (ANOVA), followed by unpaired t-test with Bonferroni correction to isolate specific differences. A value of p <0.05 was considered significant. For exercise studies, analysis of co-variance was used to test for differences between animals within groups. Mean slopes of each group were then compared using Tukey’s test.

For myo-mechanical analysis, variables for twitch, tetanus and fatigue were first subjected to a nested ANOVA to evaluate whether left and right leg measurements associated with each genotype (WT vs. KO) and age (Young vs. Aging) were similar. If so, then the left and right leg variables of an animal were averaged and the genotype and age groups were evaluated by two-way ANOVA. For the tetanus analysis, a repeated measure ANOVA was performed on the basis of the stimulus frequency of contraction (30, 60, 100, 160 Hz), which included interactions between the genotype, age and frequency. The fatigue analysis included time of maximal contraction (0.5, 1, 1.5, 2-10 min in 1 min increments). If significant differences were present between the means of groups, a multiple comparison test was performed utilizing multilple t-tests with the number of comparisons adjusted by Bonferroni correction. A value of p <0.05 was considered significant.

## RESULTS

***Morphometric analysis of hindlimb muscle from wild type and Sca-1 KO mice.***To examine the effects of Sca-1 on body mass and muscle homeostasis over the lifespan, we evaluated 12-16 week-old adult Sca-1^-/-^ (KO) and Sca-1^+/+^ (WT) mice compared with 47-50 week-old aging KO and WT mice. There was no significant difference in body weight (BW) or hindlimb weight (HW) between KO and WT mice at either age (KO young vs. WT young: BW 29.7±0.9 vs. 27.6±1.7 gms, p>0.05, HW 0.41±0.06 vs. 0.49±0.06 gms, p>0.05; KO aging vs. WT aging: BW 28.1±2.1 vs. 28.7±3.4 gms, p>0.05, HW 0.46±0.06 vs. 0.40±0.09 gms, p>0.05), or between young and aging mice (KO young vs. KO aging: BW 29.7±0.9 vs. 28.1±2.1 gms, p>0.05, HW 0.41±0.06 vs. 0.46±0.06 gms, p>0.05; WT young vs. WT aging: BW 27.6±1.7 vs. 28.7±3.4 gms, p>0.05, HW 0.49±0.06 vs. 0.40±0.09 gms, p>0.05) (**Fig. 1**). We also compared volumes of tibialis anterior (TA) and extensor digitorum longus (EDL) muscles between groups with unbiased stereology using the Cavalieri method. This demonstrated no significant difference between young and aging mice (TA-KO young vs. KO aging: 16.8±2.3 vs. 19.7±1.3 mm3, p>0.05; WT young vs. WT aging: 20.3±0.4 vs. 16.2±2.2 mm3, p>0.05; EDL-KO young vs. KO aging: 9.8±2.2 vs. 10.6±1.8 mm3, p>0.05; WT young vs. WT aging: 7.3±2.0 vs. 8.1±1.3 mm3, p>0.05), or between genotypes at either age (TA-KO young vs. WT young: 16.8±2.3 vs. 20.3±0.4 mm^3^, p>0.05; KO aging vs. WT aging: 19.7±1.3 vs. 16.2±2.2 mm^3^, p>0.05; EDL-KO young vs. WT young: 9.8±2.2 vs. 7.3±2.0 mm^3^, p>0.05; KO aging vs. WT aging: 10.6±1.8 vs. 8.1±1.3 mm^3^, p>0.05) (**Fig. 2**).

**Body and hindlimb mass in wild type and Sca-1 KO mice.**
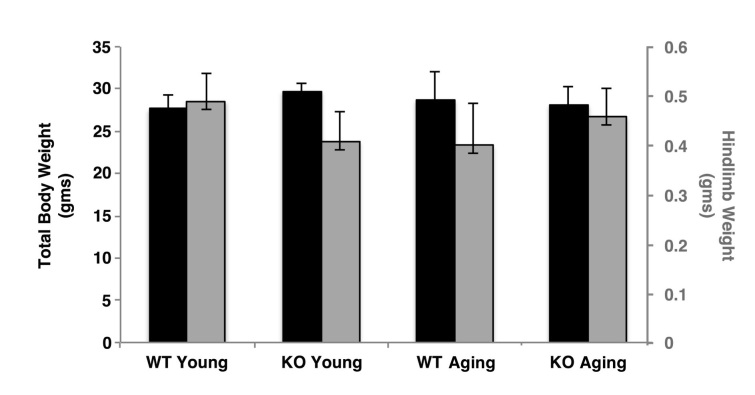
Animals were weighed immediately following euthanasia. Then hindlimbs were severed at the knee and ankle joints and weighed. No significant differences were observed between genotypes or ages.

**Hindlimb muscle volume in wild type and Sca-1 KO mice.**
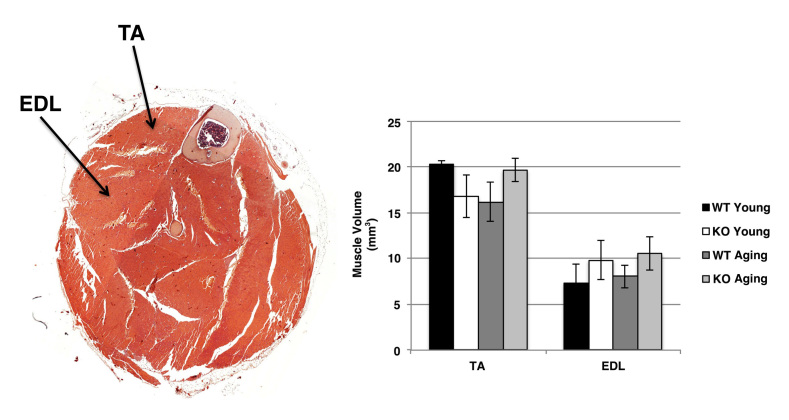
Sections were stained with hematoxylin and eosin. A typical hindlimb section is shown (left). Unbiased stereology was used to measure muscle volumes by the Cavalieri method (right). Data shown are mean±s.e.m. (N=6 hindlimbs). No significant differences were observed between genotypes or ages. EDL, extensor digitalis longus; TA, tibialis anterior.

We previously had demonstrated that Sca-1 regulates the tempo of myoblast expansion during secondary myogenesis following injury, and that regenerated KO muscle tissue is hyperplastic compared with wild type regenerated tissue 21 . To determine whether there was a difference in myofiber size between genotypes and at different ages, we compared the TA, EDL, and soleus (Sol) myofiber cross-sectional areas and perimeters between KO and WT mice at either age, or between young and aging mice. We observed that myofibers in young KO mice had greater cross-sectional areas (TA-KO vs. WT: 0.13±0.001 vs. 0.06±0.003 nm^2^, p<0.01; EDL-KO vs. WT: 0.18±0.003 vs. 0.06±0.004 nm^2^, p<0.01; Sol-KO vs. WT: 0.11±0.002 vs. 0.06±0.004 nm^2^, p<0.01) and perimeters (TA-KO vs. WT: 11.2±1.2 vs. 8.8±0.3 nm, p<0.05; EDL-KO vs. WT: 14.2±1.3 vs. 8.6±0.7 nm, p<0.01; Sol-KO vs. WT: 10.4±1.2 vs. 8.5±0.9 nm, p<0.05) than young WT mice, but that this difference reversed with aging (cross-sectional areas/TA-KO vs. WT: 0.04±0.002 vs. 0.05±0.001 nm^2^, p<0.05; EDL-KO vs. WT: 0.03±0.006 vs. 0.06±0.0.002 nm^2^, p<0.05; Sol-KO vs. WT: 0.04±0.006 vs. 0.06±0.001 nm^2^, p<0.05; perimeters/TA-KO vs. WT: 5.5±0.5 vs. 7.9±0.7 nm, p<0.05; EDL-KO vs. WT: 5.2±0.2 vs. 8.5±0.7 nm, p<0.05; Sol-KO vs. WT: 6.3±0.3 vs. 8.4±0.8 nm, p<0.05) (**Fig. 3**).

**Hindlimb myofiber dimension in wild type and Sca-1 KO mice.**
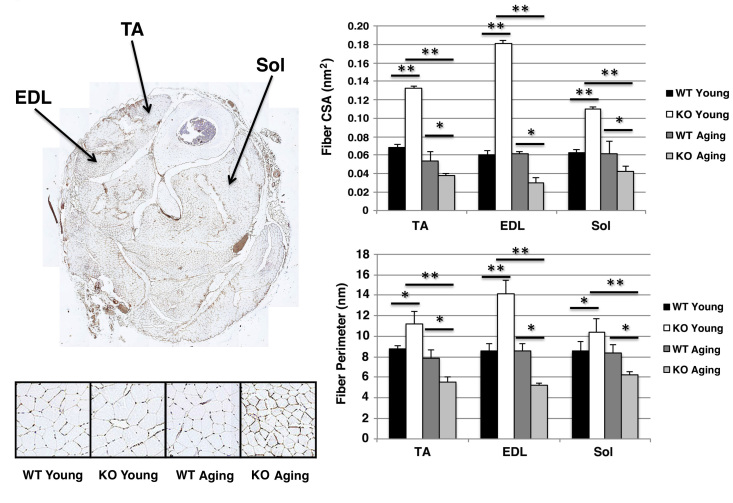
Sections were stained with anti-laminin antibody (brown) and nuclear counterstaining with DAPI (blue). A typical hindlimb section (top left), and representative micrographs (10X) from tibialis anterior (TA) muscle (bottom left) are shown. Muscle fiber cross-sectional areas (CSA; top right) and perimeters (bottom right) were measured. Data shown are mean±s.e.m. (N=6 hindlimbs). While KO young myofibers were larger in all muscles examined than WT young fibers, KO aging myofibers were smaller than WT aging fibers. *, *p*<0.05; **, *p*<0.01; EDL, extensor digitalis longus; TA, tibialis anterior; Sol, soleus.

***Satellite cell number in wild type and Sca-1 KO mice.***To determine whether there is an age-dependent difference in the number of resident satellite cells between KO and WT mice, we examined the number of Pax7^+^ nuclei localized to the satellite cell compartment in the TA muscles of KO versus WT, young versus aging animals by unbiased stereology. There was a trend toward an increase in Pax7 number in young KO versus WT muscle (KO vs. WT: 0.034±0.007 vs. 0.025±0.005 Pax7^+^/DAPI^+^ nuclei, p>0.05), this was not significant, and this difference decreased in magnitude in older animals (KO vs. WT: 0.026±0.003 vs. 0.030±0.001 Pax7^+^/DAPI^+^ nuclei, p>0.05) (**Fig. 4**).

**Pax7+ cells in hindlimbs of wild type and Sca-1 KO mice.**
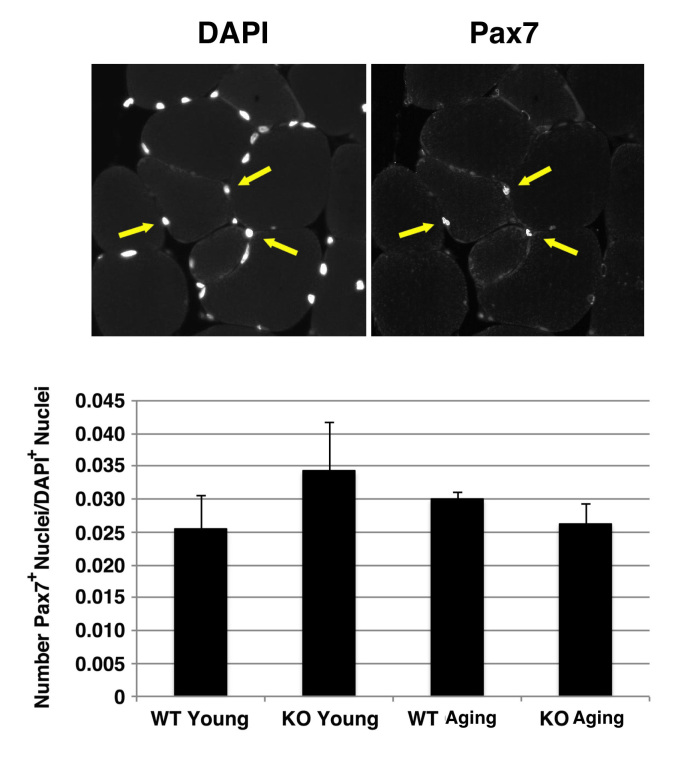
Sections were stained with anti-Pax7 antibody. A typical micrograph is shown (20X); arrows indicate Pax7^+^DAPI^+^ nuclei (top). Data shown are mean±s.e.m. (N=4 hindlimbs) (bottom). No significant differences were seen between genotypes or ages.

***Vascularity in hindlimb muscle of wild type and Sca-1 KO mice.***Sca-1 is expressed on the surface of a variety of stem cells 24 , including vascular endothelial progenitors 25 . To determine whether muscle vascularity was affected by Sca-1 expression, we compared the number of arterioles and the total arteriolar cross-sectional area in TA muscles of young versus aging, KO versus WT mice. We found that while the number of arterioles increased with age (KO young vs. KO aging: 28.8±6.7 vs. 81.6±10.5 SMA^+^CD31^+^ vessels/section, p<0.001; WT young vs. WT aging: 30.2±4.0 vs. 70.29.5 SMA^+^CD31^+^ vessels/section, p<0.001), the total arteriolar cross-sectional area decreased (KO young vs. KO aging: 590.5±116.1 vs. 264.4±88.5 µm2, p<0.01; WT young vs. WT aging: 569.1±72.0 vs. 259.0±72.9 µm2, p<0.01) (**Fig. 5**). There was no difference, however, between KO and WT mice (KO young vs. WT young: 590.5±116.1 vs. 569.1±72.0 µm2, p>0.05; KO aging vs. WT aging: 264.4±88.5 vs. 259.0±72.9 µm2, p>0.05) (**Fig. 5**).

**Hindlimb vascularity in wild type and Sca-1 KO mice.**
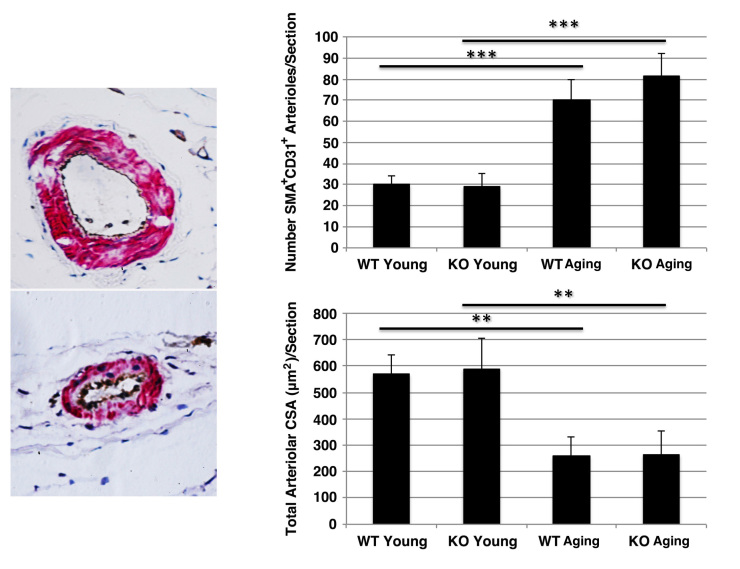
Sections were stained with anti-SMA (brown) and –CD31 (pink) antibodies. Typical micrographs are shown (40X) (left). A significant increase in the number of vessels, accompanied by a decrease in total vascular cross-sectional area (CSA) was observed with age, however, this was independent of genotype (right).

***Fibrosis and myoblast proliferation in hindlimb muscle of wild type and Sca-1 KO mice.***Previously, we had shown that KO myoblasts activated during the response to injury have a prolonged proliferative phase with resulting hyperplasia during healing 21 . To determine whether muscle homeostasis, or secondary myogenesis with normal use, resulted in a difference in fibrosis with age, we compared the extent of fibrotic tissue in cross-sections from young and aging, KO and WT hind limb muscle following a 37 day course of voluntary and forced exercise (described below; **Figs. 8, 9**). We found no difference between genotypes or at different ages (**Fig. 6**). To determine whether upregulated proliferation during secondary myogenesis in KO animals leads to exhaustion of proliferating myoblasts with age, we compared the number of Ki67^+^ proliferating cells in the TA muscles of these mice, and found no significant differences (**Fig. 7**).

**Hindlimb fibrosis in wild type and Sca-1 KO mice.**
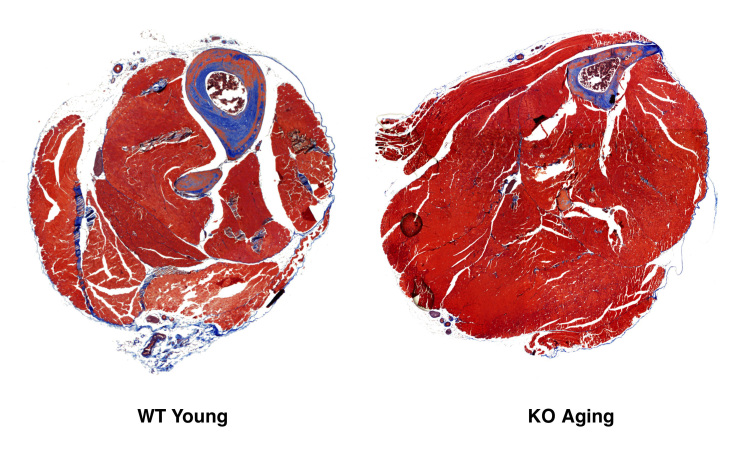
Hindlimbs were skinned, fixed and embedded in paraffin, and sectioned prior to staining with Masson’s trichrome. Typical hindlimb sections are shown; muscle and intercellular fiber (red), collagen (blue), nuclei (black). Unbiased stereology was used to measure scar (blue) volumes in all genotypes and ages by the Cavalieri method (data not shown). No significant differences were observed between groups.

**Ki67+ cells in hindlimbs of hindlimbs of wild type and Sca-1 KO mice.**
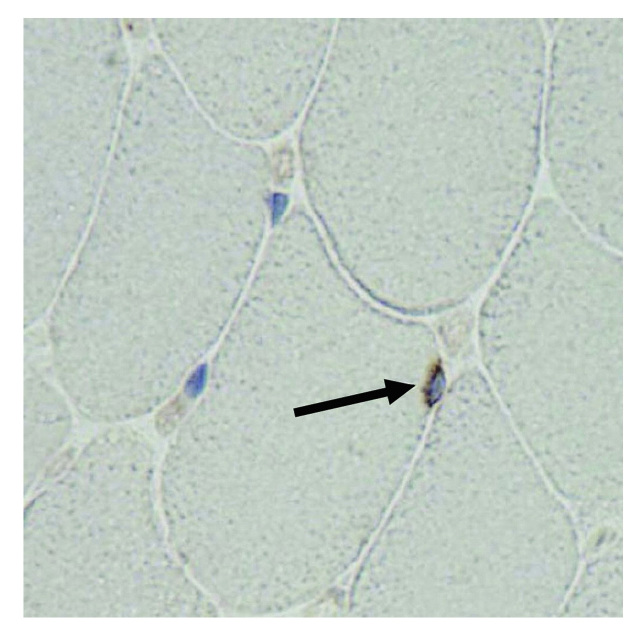
Hindlimbs were skinned, fixed and embedded in paraffin, and sectioned prior to immunohistochemical staining with anti-Ki67 antibody (brown) and DAPI (blue). A typical micrograph is shown (60X oil); arrow indicates Ki67^+^DAPI^+^ nucleus. No significant differences were seen between genotypes or ages.

***Exercise capacity in wild type and Sca-1 KO mice.***To assess the functional significance of the difference in myofiber size between young and aging KO versus WT mice, we evaluated exercise capacity of these animals during voluntary (**Fig. 8**) and forced (**Fig. 9**) exercise. Mice were allowed to run at will during normal light-dark cycles over consecutive 24h periods. For voluntary distance, analysis of co-variance demonstrated no statistically significant differences between animals within any group, but the mean slopes of all groups (Day versus Distance: WT young 64.4, KO young 43.6, WT aging 41.9, KO aging 29.5) were statistically significantly different from each other (p<0.0001), except for WT aging and KO young, which were statistically the same (p>0.05) (**Fig. 8**). For voluntary duration, analysis of co-variance demonstrated no statistically significant differences between animals within any group, but the mean slopes of all groups (Day versus Duration: WT young 127.0, KO young 105.5, WT aging 81.5, KO aging 60.0) were statistically significantly different from each other (p<0.01) (**Fig. 8**). Voluntary exercise showed that young KO mice achieved significantly less distance and duration than their WT counterparts, and resembled aging WT animals. Similarly, aging KO mice achieved significantly less distance and duration than aging WT mice.

**Voluntary exercise in wild type and Sca-1 KO mice.**
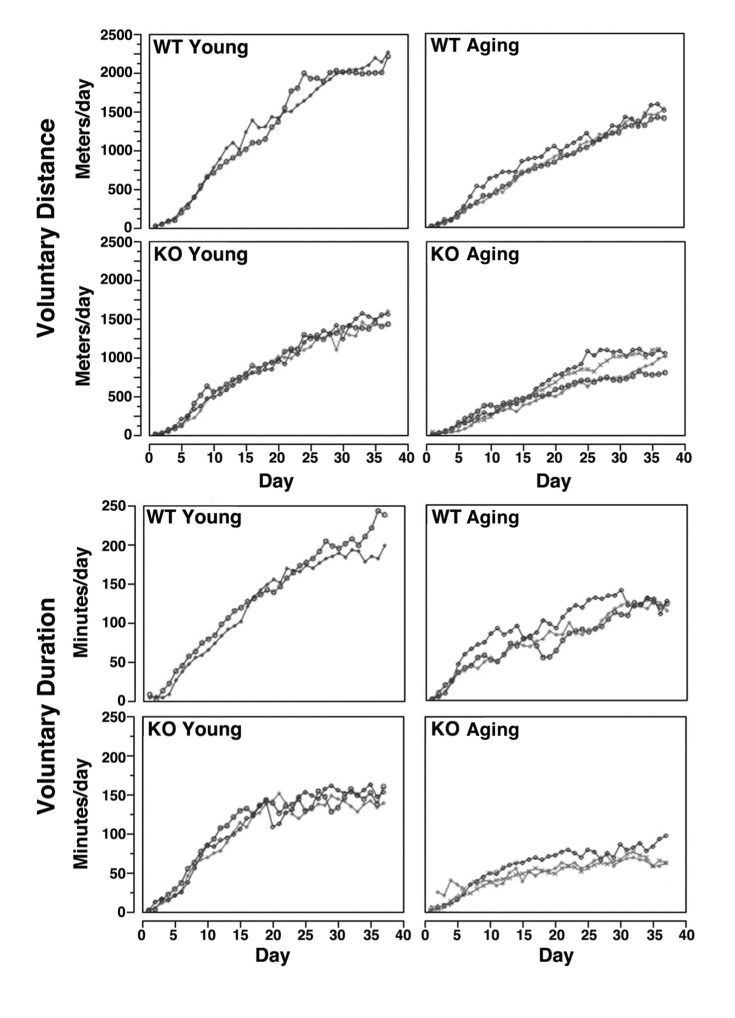
Mice were allowed to run at will during normal light-dark cycles. Each line represents data from one animal. Distance and duration over consecutive 24 hr periods were recorded. Distance (meters/day; top) and duration (min/day; bottom) were plotted for individual animals over a 37 d period. For voluntary distance (top), analysis of co-variance demonstrated no statistically significant differences between animals within any group, but the mean slopes of all groups (Day versus Distance) were statistically significantly different from each other as determined using Tukey’s test, except for WT aging and KO young, which were the same. For voluntary duration (bottom), analysis of co-variance demonstrated no statistically significant differences between animals within any group, but the mean slopes of all groups (Day versus Duration) were statistically significantly different from each other as determined by Tukey’s test.

On each day over a 37d period, mice also were forced to run using a protocol where speeds were progressively increased until mice reached their maximum rate. Analysis of co-variance demonstrated no statistically significant differences between animals within any group, but the mean slopes of all groups (log(Day) versus Maximum Rate^2^: WT young 45.6, KO young 41.1, WT aging 32.4, KO aging 21.6) were statistically significantly different from each other (p<0.01) (**Fig. 9**). Forced exercise similarly demonstrated that young KO mice achieved a lower maximum exercise rate than young WT mice, as did aging KO animals compared to aging WT controls.

**Forced exercise in wild type and Sca-1 KO mice.**
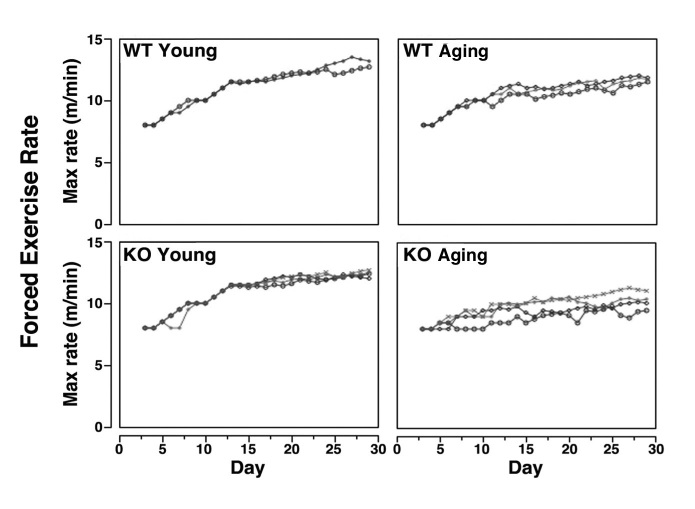
On each day over a 37 d period, mice were forced to run at 8 meters/min for five 10 min intervals with 30 sec rests between intervals. Over the next five 10 min intervals immediately following that day’s exercise session, speeds were progressively increased until mice reached their maximum rate, as determined by the maximum speed (meters/min) at which mice continued to run on the wheel without falling off or hanging onto the wheel. Each line represents data from one animal. Analysis of co-variance demonstrated no statistically significant differences between animals within any group, but the mean slopes of all groups (log(Day) versus Maximum Rate2) were statistically significantly different from each other as determined using Tukey’s test.

***Myomechanical analysis of hindlimb muscle from wild type and Sca-1 KO mice.*** To quantify changes in muscle function observed during exercise, we performed myomechanical analysis of isolated EDL muscle from KO and WT young and aging mice. There were no significant differences in muscle mass or muscle cross-sectional area between ages and genotypes, consistent with data from isolated hind limbs (**Fig. 1**) and unbiased stereology assessment of muscle volumes by Cavalieri method (**Fig. 2**). Aging animals (KO aging 3.4±0.4 x 10^4^, WT aging 3.9±0.6 x 10^4^) had significantly lower muscle:body mass ratios compared with young animals (KO young 3.9±0.6 x 10^4^, WT young 5.5±0.5 x 10^4^; p<0.05), however, this difference was similar for both KO and WT mice.

Analysis of muscle twitch demonstrated that aging mice (KO aging 115±5 mN, WT aging 101±7 mN) generated higher maximal tension (Pt) than young animals (KO young 84±7 mN, WT young 89±6 mN; p<0.05), but again there was no difference between KO and WT mice. Aging mice (KO aging 23±1 ms, WT aging 24±1 ms) also had shorter contraction times (CT) than young mice (KO young 29±1 ms, WT young 25±1 ms; p<0.05), but with no genotype-specific difference. There were no significant differences in specific maximal twitch tension (sP_t_) or half-relaxation time (HRT) between animals. Analysis of tetanus revealed similar results, with an age-dependent increase in maximal tetanic tension (P_0_; KO aging 372±15 mN, WT aging 357±22 mN vs. KO young 297±23 mN, WT young 302±18 mN; p<0.05) and maximal rate of rise of tetanus (MRRT; KO aging 21.1±0.9 mN/ms^-1^, WT aging 19.5±1.3 mN/ms^-1^ vs. KO young 16.0±1.3 mN/ms^-1^, WT young 17.5±1.0 mN/ms^-1^; p<0.05). There were no significant differences in specific maximal tetanic tension (sP_0_) or P_t_/P_0_ ratio between animals.

Force-frequency analysis of isolated EDL muscle from young and aging KO and WT mice showed no significant difference between genotypes or ages (**Fig. 10** *left*). A low-frequency fatigue protocol, however, showed a significant difference between KO young muscle and all other groups (p<0.05), with KO young muscle generating a greater percent of maximal force, and less fatigue, at all time points over 10 min (**Fig. 10** *right*). We also examined the percent maximum force generated with relaxation during low-frequency fatigue, to determine whether there was any difference in the return to baseline between contractions between the groups. Although the force generated during relaxation trended upward over time (i.e., incomplete relaxation with fatigue), this showed no significant differences between groups (**Fig. 10** *right*).

**Ex vivo myomechanical analysis of EDL muscle from wild type and Sca-1 KO mice.**
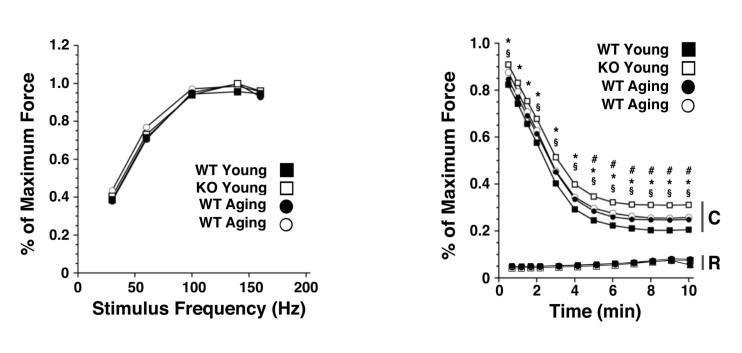
Extensor digitorum longus muscle was dissected, mounted in a force transducer, and stimulated as decribed in Methods. Data shown are mean percent maximum force (N=12 young WT; N=2 young KO; N=3 aging WT; N=9 aging KO). No differences were observed in force-frequency relationships between genotypes or ages (left). Young KO animals demonstrated significantly greater maximum contraction (C) force (less fatigue) over time compared with other groups, although relaxation (R) force was the same between groups (right). Absolute peak force at the beginning of the study was statistically similar for all groups (KO Young 198±40 mN, KO Aging 234±66 mN, WT Young 208±52 mN, WT Aging 186±30 mN). *, p

## DISCUSSION

While previous studies have provided focused investigations of Sca-1 KO animals 6 ^,^ 10 ^,^ 11 ^,^ 20 ^,^ 21 ^,^ 26 , this represents the first comprehensive analysis of the tissue, mechanical, and functional effects of Sca-1 gene disruption on murine skeletal muscle. Compared with wild type animals, we found that myofiber size was significantly increased in young KO mice, but then significantly decreased with age. Consistent with this difference, we observed greater force generation in young KO hindlimb muscle versus wild type or aging KO muscle. Interestingly, both young and aging KO animals demonstrated decreased conditioning with exercise compared with wild type littermates.

Our observations of myofiber area and perimeter are similar to another study that demonstrated a 19% increase in mean cross-sectional area in 2- to 4-month-old Sca-1 KO TA muscles, and a 13% decrease in mean cross-sectional area in 1-year-old Sca-1 KO TA muscles 20 . Those authors suggested that this difference may be the result of differences in satellite cell number or function, however, we saw no alteration in the number of Pax7^+^ cells beneath the basal lamina of WT versus KO or young versus aging muscles. In addition, we observed no difference in the number of proliferating, Ki67^+^ cells between various groups, suggesting that the difference in myofiber size with genotype or age is not due to alterations in number of satellite cells or proliferating myoblasts. More recently, Kafadar et al. showed that Sca-1 KO muscle displays reduced matrix metalloproteinase activity, suggesting that the effect of Sca-1 on myofiber size may be mediated through extracellular matrix interactions 26 .

Sca-1 is normally expressed on vascular endothelial progenitor cells 25 , so we also wanted to evaluate whether an alteration in vascularity in Sca-1 KO muscle might be responsible for the difference in myofiber development in young KO animals. However, we found no difference in vascular cross-sectional area or vessel number between WT and KO animals at either age, although a shift from fewer, larger vessels to more numerous, smaller vessels with age was observed independent of Sca-1 expression. We did not directly examine vascularity or composition of the myocardium in Sca-1 KO animals. We recently showed that Sca-1-expressing cells differentiate into multiple myocardial cell types in vitro, and that these cells induce angiogenesis and differentiate into endothelial and smooth muscle cells in mouse hearts following myocardial infarction 27. It is possible that Sca-1 deficiency in KO animals also may be playing a role in physical conditioning as a function of cardiovascular fitness.

Analysis of twitch and tetanic tension generated by Sca-1 KO muscle compared with WT showed no significant differences. In addition, the response to forced-frequency fatigue was preserved in KO as well as older muscle. However, in the case of time-dependent fatigue at low frequency stimulation, we did observe that young KO muscle generated greater force at most stimulation frequencies compared with young WT or aging WT and KO muscle. This coincided with an increase in myofiber size seen in young KO muscle. Interestingly, the relative decrease in myofiber size in aging KO animals did not lead to a downward shift in the force-frequency curve. It is possible that the myofiber environment may play a greater role in myomechanical function than currently presumed. The positive regulatory effects of Sca-1 signaling on matrix metalloproteinases has been observed in regenerating muscle (26). Further investigation into the impact of Sca-1/extracellular matrix interactions on myomechanical function are needed.

Our experiments showed that the absence of Sca-1 affected exercise performance *in vivo*, and that age compounded these effects. Young KO mice exercised for shorter time periods and ran shorter distances during voluntary exercise, and achieved slower maximal exercise rates during forced exercise, than young WT mice, and achieved results similar to aging WT animals. Aging KO mice showed an even greater decline in voluntary and forced exercise performance. In addition, KO mice did not improve with practice, while WT animals demonstrated improved performance over time. These findings suggest that although Sca-1 KO did not negatively impact muscle strength ex vivo, Sca-1 expression contributes to the response to exercise, and that the subnormal response observed in Sca-1 KO animals becomes more pronounced with age.

## COMPETING INTERESTS

The authors have declared that no competing interests exist.
